# Perspectives of agriculture, nutrition and health researchers regarding research governance in Malawi. Using a leadership, ethics, governance and systems framework

**DOI:** 10.1186/s12910-023-00940-x

**Published:** 2023-08-21

**Authors:** Limbanazo Matandika, Kate Millar, Eric Umar, Joseph Mfutso-Bengo

**Affiliations:** 1 Center for Bioethics in Eastern and Southern Africa (CEBESA), Kamuzu University of Health Sciences (formerly known as the University of Malawi, College of Medicine), Private Bag 360, Blantyre, Malawi; 2 Centre for Applied Bioethics, Schools of Biosciences and Veterinary Medicine and Science, University of Nottingham, Sutton Bonington Campus, Loughborough, LE12 5RD UK; 3 Health Systems and Policy Department, Kamuzu University of Health Sciences (formerly known as the University of Malawi, College of Medicine), Private Bag 360, Blantyre, Malawi

**Keywords:** Agriculture Ethics, Ethical competency, LEGs Framework, Research Governance, Malawi, Research Ethics Committee

## Abstract

**Background:**

Research ethics is intertwined with and depends on building robust and responsive research governance systems alongside researchers. Globally there has been substantial investment in agriculture, nutrition, and health (ANH) research motivated by the need to improve health outcomes, such as micronutrient deficiencies in Sub-Saharan Africa. Although there has been a notable focus on ethical issues inherent in ANH studies, there has been scanty research examining researchers’ attitudes related to ANH research. This study was conducted to explore the perspectives of researchers who conducted an agronomic biofortification study in Malawi.

**Methodology:**

In-depth interviews were conducted with a purposive sample of ten ANH researchers. Interviews were conducted online via Zoom, audio-recorded, transcribed verbatim, and thematically analysed using the Leadership, Ethics, Governance and Systems Framework.

**Results:**

Four core aspects emerged: Leadership: The relevance of building ethics leadership and ethical competence among researchers. Ethics: There is a need to develop a framework that operationalises core ethical values that can guide the implementation of ANH research. Governance: Research guidelines were perceived to be too generic to guide ANH research. Systems: Researchers’ recommended the establishment of a specialised ANH research ethics committee.

**Conclusions:**

The findings highlight the significance of building ethics leadership and supporting ethical competency amongst researchers. Researchers recommended the development of tailored approaches rather than utilising generic governance systems and frameworks that are drawn from medical research and thus not fit for purpose in this field. In Malawi, specialised ethics review committees are needed to guide ANH research.

**Supplementary Information:**

The online version contains supplementary material available at 10.1186/s12910-023-00940-x.

## Background

There has been substantial investment in agriculture, nutrition, and health research (ANH). One area of focus is the need to improve micronutrient deficiencies in Sub-Saharan Africa [1–3]. As these studies increasingly involve human participants or cut across the agriculture and human health nexus, they are ethically significant research activities. To enhance study participants’ protection, rights, and well-being, research practices in this area are guided by ethical principles [4]. Furthermore, to ensure compliance with applicable ethical standards, research recruiting human participants requires independent ethics committee review [5]. Although there has been a notable focus on ethical issues raised by this area of research and the importance of protecting participants, research regulations that specifically govern the conduct of newer research fields like ANH are lacking [6]. Researchers have expressed concerns regarding the nature and extent of the research review process with no due consideration of evolving methods, underpinning ethics, and values [7]. There is a lack of specific research policies, guidelines, institutional capacities to coordinate research, and strategic plans [6, 8–10].

Ethical issues and dilemmas occur in all forms of research when human participants are involved [11] and this is no less the case within ANH research. Ethical competence is the capacity that researchers need to possess to identify those dilemmas and ethically handle them [12–14] and such competence is also a crucial factor that enables researchers to resolve ethical dilemmas, make value-based decisions and implement ethically sustainable good research practices [15]. It is important to raise and improve ethical awareness, the acquisition of ethical knowledge, and support reflection on professional practice [16]. These studies demonstrate challenges in the governance and oversight of research in Africa in general but also highlight the need to explore attitudes, knowledge, and practices among researchers conducting research in newer fields.

The field of ANH is committed to innovative research investigating public health interventions to alleviate nutritional deficiencies for example selenium through agro-bio fortification [3, 17]. It is therefore important to identify the opportunities and challenges of providing robust ethical governance in this field. There is a need to examine the attitudes of ANH researchers towards concepts of research ethics, including research leadership, ethical consideration, governance, and systems. This work would appear timely as researchers have recently expressed the need to develop ethical frameworks that would govern the implementation of nutrition-related public health interventions [18]. This study, therefore, aims to characterize and examine ANH researchers’ knowledge, awareness, and attitudes regarding ANH research governance in Malawi. As a result, the **L**eadership, **E**thics, **G**overnance and **S**ystems ( LEGS) Framework has been used to structure and support the analysis of the perspectives of the ANH researchers.

## LEGS framework

Data collection and analysis were guided by the LEGS framework proposed by Mfutso-Bengo [19]. The tool was originally designed for designing and developing resilient and responsive health governance systems. The framework’s four pillars include; leadership, ethics, governance, and systems. The first pillar of leadership involves ethical competence with its pinnacle broadening on the education aspect that enhances moral imagination. Ethics includes an investment in promoting the practice of virtues and moral reasoning skills. Ethical considerations and ethical decision making ought to be guided by four ethical principles, namely: (i) Beneficence: “Do good” and (ii) Non-Maleficence (“do no harm”) enact the obligations of researchers to ensure that anticipated benefits are realised and anticipated risks are minimized., (iii) respect;for persons; participants should be treated as autonomous agents and their choices be respected and (iv) justice; there be equal distribution of benefits and risks in research and if there is unequal treatment it be justified. Principles and values that uphold the conduct of research in the field should be considered. *Governance* involves ensuring the existence of research frameworks, regulations and guidelines with attention to oversight and monitoring. A LEG frameworkis regarded as a preceding building block that requires a *system a*s a proceeding block to achieve its intended goals. Systems in research governance involve the existence of an ethics review committee to ensure that the ethical standards and scientific merit of research involving human subjects are met.

## Methods

### Study design and research participants

This cross-sectional qualitative study was conducted from February 2021 to May 2021.

We recruited ten researchers (soil researchers, nutritionists, farm managers, geochemists, plant nutritionists, statisticians) and team members of the Addressing Hidden Hunger with Agronomy (AHHA) trial, which was part of a wider funded project examining opportunities and challenges of agro-bio fortification in Southern Africa [17].

### The AHHA trial

The AHHA trial was conducted in 2019 in Kasungu district in the central region of Malawi and published [17]. The project was a collaboration between the Lilongwe University of Natural Resources (LUANAR) and the Kamuzu University of Health Sciences (formerly known as the University of Malawi, College of Medicine) in Malawi, the University of Nottingham and the London School of Hygiene & Tropical Medicine in the UK. It was a community-based randomised controlled trial to address micronutrient deficiencies (hidden hunger) through agro-bio-fortification, which is widespread in Malawi. A national survey revealed 62.5% of women of reproductive age (WRA; n = 802) had plasma Se concentrations below a threshold for the optimal activity of the Se-containing protein glutathione peroxidase 3 (GPX3; <84.9 µg L − 1) which plays a role in antioxidant function [3]. A small concentration of Se in maize grain (median 0.0188 mg kg − 1, n = 1,806) is found throughout most of Malawi (10), resulting in inadequate dietary Se intakes leading to widespread Se deficiency measured in blood and urine [1]. The trial sought to test the efficacy of improving selenium status in women and children through the consumption of selenium-agro-fortified maize flour. Agro-fortification involves enriching a food vehicle with a micronutrient during crop production, e.g., through the use of fertilisers [1, 17]

The trial randomised 180 households each contributing one participant women of reproductive age (WRA, 20–45 years of age) and one school-aged child (SAC, 5–10 years of age) to receive maize flour enriched with selenium (n = 90 households) or not enriched (control; n = 90 households) [20]. A total of 180 households participated in the trial with households receiving enough flour to meet all their constituent member needs (i.e. 330 g/capita/day) for 8 weeks. The study additionally provided maize flour (not enriched with selenium) to all other households in the study area to reduce the likelihood that participant households sold or gifted away their allocated flour [21]. The study activities included anthropometry, blood sampling and dietary assessment at baseline, distribution of the study flour during the intervention and adherence monitoring.

The AHHA trial protocol and amendments were approved by the London School of Hygiene & Tropical Medicine Interventions Research Ethics Committee (reference: 16,181) and the Malawi College of Medicine Research Ethics Committee (reference: P.11/18/2539), and the trial was a registered clinical trial (March 2019; ISCRTN85899451 [17].

### Sampling and selection of study participants

This study used purposive sampling to select study participants who were involved in the design, implementation or reporting of the AHHA trial. We selected ten AHHA Malawi trial team members for in-depth interviews (IDIs). This approach enabled the researchers to collect in-depth information based on participant’s overall trial experiences, knowledge, and exposure [22]. All selected trial team members were contacted through email as an introduction to the aim of the study. The lead bioethics researcher (LBR) then set up a zoom or phone interview. An information sheet and informed consent form were sent to those who consented. Informed consent forms were sent to researchers a day before the interview. The LBR conducted all the IDIs in the study.

### Data collection tools

We developed an IDI guide consisting of open-ended questions guided by the LEGS framework (Additional file 1). The IDI guide topics asked the researchers to comprehensively illuminate their perspectives on their research work with questions linked to the four aspects of the LEGS framework. The interviews were automatically digitally recorded and a secondary recorder was used as a backup in case the Zoom call recording failed. All interviews were conducted in English and lasted between 40 and 55 min. After interviewing eight researchers, the study reached data saturation, and the BLR collected no new fundamental insights. However, we conducted two more interviews to validate the data saturation [22].

### Data management and analysis

The interviews were transcribed verbatim by an independent transcriber. After each interview, the BLR sent the recorded audio to the transcriber electronically, the audios were secured with a password and were encrypted. The transcripts were shared amongst the research team and the LBR cross-checked all transcripts for consistency with the original recordings. The recorded audios and transcripts were kept on a password-protected computer that could only be accessed by the BLR. Coding used deductive descriptive codes (see Table 1) generated from the LEGS framework by Mfutso-Bengo et al., (2017), with the data managed using NVivo 12.0.

The data were analysed using a thematic analysis framework [23], which allowed deductive coding with the guidance of the LEGS framework. Codes were organised into themes per the framework, and subthemes that emerged were categorised into the main themes. The LBR coded the transcripts which were then reviewed by the two bioethicists, and further discussed to agree on the codes and subthemes.

### Ethical considerations

This study was approved by the College of Medicine Research and Ethics Committee (COMREC) on 21 May 2019. Its ethics reference number is P.03/19/2633.This study was originally reviewed by COMREC 2 years before data collection. The revised research tools were reviewed through an amendment that was approved on 16 Dec 2020. All participants were assured that their participation was voluntary and written informed consented was obtained before the interviews. Participants were reminded that they could withdraw at any time up until publication and the interview would be recorded on the day.

**Table 1:** Defined themes using the LEGS framework

Framework	Main themes	Subthemes
**L**	Leadership	• Awareness of ethical issues • Capacity building, is considered through research ethics Training and research ethics review. • Support for the role of ethical reflection in research
**E**	Ethical	• Social Value • Sustainability • Community ownership
**G**	Governance	• Policies and guidelines that govern research conduct in Malawi • Supporting and Protecting the Researchers • Protecting Dignity and Rights of Participants
**S**	Systems	• Competency of ethics review of Agriculture research • Process dilemmas with a medical review of Agriculture, Nutrition and Health Research • Transparency and Accountability within the system

### Findings

The experiences and insights of the researchers are presented under the LEGS framework core areas, with a series of important themes emerging under Leadership, Ethics, Governance, and Systems.

### Leadership in research

We have classified the findings of the Leadership theme according to the following three subthemes; awareness of ethical issues, capacity building and support for ethical reflectivity. This classification provides an overview of the perspectives of the researchers on leadership.

#### Awareness of ethical issues

Researchers reported having several ethical dilemmas in ANH research including; in study designs, cultural considerations, informed consent, research knowledge and experience, power dynamics, and ethical guideline utilization. The researchers understood the importance of mapping, analysing and responding to ethical problems inherent in ANH research. Additionally, the researcher expressed a sense of ethical oblications and responsibility towards their trial participants, highlighting the importance of ethical knowledge.

*“…, I think it is about doing the right thing in as far as human interaction and in this case, in terms of research. And doing the right thing has a lot of items in it. Number one, you are not taking advantage of people you are going to interact with. There is a social interaction that is likely to be there between researchers coming from a university, arriving in a village on a vehicle. You tend naturally to have an imbalance. Where you are arriving, these people will have the power, and that is their community, and they are free to allow entry or not allow” IDI 01.*

#### Capacity building

When reflecting on how leadership can be developed and nurtured, researchers discussed the role of and need for capacity building. Within this project, the researchers had access to several research ethics training activities, including research tailored ethics training and an ethics workshop at a project level meeting. Reflecting their commitment to supporting good research practices, researchers noted that training and collaboration enhanced their knowledge of ethics. For the AHHA trial team, a research ethics training workshop was a prerequisite for all team members before the recruitment of trial participants.

*“., l think it strengthened my view, looking at this from a different dimension so we might look at that as enhancing l knew we need to do ethics but l look at it as an important dimension of embedding ethics within research as you are doing it, so it is part of the process so l will put it in that way. Enhancing, broadening my understanding of ethics as part of the research.” IDI 01*.

Collaboration with ethics experts was seen to support ethical mindfulness. Researchers stated that engagement and partnership enhanced their ethical ability to map, analyse in partnership and address ethical issues.

*“….Once the results are published, we intend to go back and that was strengthened because you have ethics people around and you promised that you will do it and so we just had to go back and it is a nice thing to do” IDI 01.*

*….” l likened that we got ethics people on this trial and it just lets you be more alert and to see, and we are a lot more conscious to see for fear that we are going to do the wrong thing. You become more alert and catch out issues so that you are doing the right thing ….” IDI 001*.

Researchers revealed that ethics knowledge sharpened their ability to be mindful of ethical issues presented by the study context, culture, and the application of ethical principles. The researcher’s attitude and adaptation of strategies in responses to their ethical analysis of issues highlighted the significance of building ethical leadership in the ANH discipline. Researchers also demonstrated the importance of capacity-building initiatives through collaboration and training in improving ethics knowledge and moral reasoning.

### Ethical issues

Several key aspects emerged which are categorised under the ethics theme, with the identification of three core subthemes namely sustainability, community participation and social value.

The ANH researchers characterised various ethical and social considerations that inform the conduct of their research. There was emphasis on how particular social and ethical values differed across researchers which reflected on their diversity of expertise, roles, and responsibilities in the trial. Those who worked directly with communities, highlighted essential aspects of ANH research that were participant-centred and need more careful consideration. Interviewees who did not interact with trial participants at the community level also discussed several core values.

#### Sustainability

All researchers mentioned the importance of sustainability in all ANH research interventions. Researchers defined sustainability as the ability of community members to continue implementing various ANH research interventions in their communities after research has ended.

“… *Well, on sustainability in the context of Agricultural Research, Agricultural projects or intervention projects, have to do with the continuation of the benefits from the projects rather be elements that are learned from the projects, long after the project itself has ended”* IDI 04.

Researchers also highlighted the importance of ensuring that communities have taken total ownership of interventions in discussing sustainability. Another critical aspect of suitability included the ability to build capacity at the local level.

#### Community participation

As revealed by the ANH research, community involvement and knowledge play a crucial role in ensuring a community’s acceptance and ownership of interventions. Researchers indicated that the involvement of community members results in a “win-win” approach where the researcher collects new data whilst community members benefit when the intervention has proven effective.

*“…. sustainability is people continuing with what they’ve learned long after the project has ended while ownership is the beneficiaries of the project are meant to have a sense of, or rather see a sense of ownership within the project, so where they believe and understand that the project is for them and their benefit and not necessarily just a means to an end from the organisation that is implementing it. These two are interrelated, the projects that have a high sense of ownership means there’s a higher level of engagement from the beneficiaries because this is their thing and they want to embrace it, and they want to fully support and be proud of it because I am guessing they understand the importance and the necessity” IDI 04*.

#### Social value

Another value that was emphasized by the researchers was the construction of social value, with researchers highlighting that the intervention should meet the needs of the local communities, and local communities should deem it beneficial. Other researchers felt social value is about the acceptability of the intervention.

*“… If it is a technology, then l want to leave that experience with them, they may like it, and they may decide whether it works or does not for them.” IDI 01.*

Based on the ethics theme, perspectives centered on the (i) the ability of an intervention to generate knowledge that leads to improvement of health in the community, (ii) ability of community members to use the knowledge or intervention. In addition, community members should use the knolwegde beyong the study period.

### Governance

Issues of governance emerged and researchers discussed policies and guidelines that govern the conduct of ANH research, particularly emphasizing the governance structure for research conducted in Malawi.

#### Policies and guidelines that govern ANH research in Malawi

Research guidelines were seen to promote ethical and social values. The researchers were aware of the policies governing the conduct of research in Malawi, such as Malawi’s guidelines for conducting research. Researchers acknowledge that ANH is not a new field, but research regulation is not as well established compared to clinical research. Researchers hinted at the lack of discipline-specific ethical frameworks.

“…*I can share my experience…That agriculture nutrition research is not a new field but perhaps as not well established and not as large as research areas such as infectious diseases, vaccines, and that kind of thing. So in terms of ethics vs protocol frameworks, l feel that reviewers starting from scratch doing everything with the agriculture nutrition ethics research community and things look a bit different and there is some adaptation you need to do.”* IDI 07.

Researchers also highlighted how diverse the ANH field has grown over the years, advocating for the need to have well-established guidelines for its activities. The researchers also highlighted how challenging it is to oversee some research activities, for example, animal research.

“….*l have been talking to our leaders in the directory of research and outreach and say let’s have an internal research ethics committee/ guidelines because it would have varied membership, people that can understand the issues that are there in pure agriculture, pure nutrition and then in between where these are mixed because there are quite varied, so that is one of my worries and l am sure that once at* the research institute *we have, what is this, vet medicine and now ethics with animals is an issue. The other day we had a conversation that looks here now we have vets, it is no longer just human subjects but also livestock and other animals that we need to be thinking about, quite varied and then we have environmentalists thinking about what we are doing to the environment. […] now is very comprehensive and we need a comprehensive kind of research ethics board. The one that we go to, the virtue of being in the ministry of health, l think they are more inclined to clinical research* IDI 08.

Researchers acknowledged that current guidelines that may be designed for medical studies failed to be supportive in some circumstances but rather appeared to be narrow or non-specific or at times did not acknowledge the norms in their fields like agriculture. Some researchers said they experienced dilemmas after weighing recommendations from research guidelines against the risk of violating core norms upheld in the ANH field. Dilemnas included whether to compensate or not. Based on Malawi’s agriculture good research practises, monetary compensation is prohibited.

### Supporting and protecting the researchers

Researchers acknowledged the availability of general research guidelines as a critical enabling factor and hinted that research guidelines offer a hands-on resource when designing trials. They offer practical information on how to design informed consent such as the framework of guidelines for research in the social sciences and humanities in Malawi. Researchers reported that guidelines helped them to conduct the study per the relevant legal and ethical regulatory requirements.

### Supporting and protecting the rights of participants

Researchers discussed the value of the governance approaches for protecting participants, which matched their values. However, the most notable limitation of the national guidelines was that the researchers felt that they are oriented and written from the perspective of doing research in medical and hospital settings. This was because there was minimal guidance on animal research, the environment, and operational agri-nutrition research within communities.

..*It was good l felt l was involved in the trial with an understanding and provided very helpful practical guidance on other issues that are often encountered when running these kinds of studies like participant compensation, and informed consent processes. IDI 07*

### Systems

A further three subthemes emerged from the interviews which relate to systems in which the research operates and systems which are designed to support research. Specific subthemes emerged were, significance of ethics review, dilemnas from medicial ethics framing during review of ANH research, and transparency and accountability within the system.

#### Ethics review of ANH research

All researchers agree that an ethical review system is essential for ANH research. Furthermore, undergraduates and postgraduates apply for ethics approval through the National Health Science Research Ethics Committee (NHSRC) and the COMREC in Malawi along with ANH researchers. Researchers understood Malawi’s research ethics committee roles.

***“….***, *I have understood the importance of ethics quite a lot. All the students, undergraduate and postgraduate, for us at Research Institute, make ethics applications through the national science research ethics committee which is based in the Ministry of Health. And that committee receives all kinds of research and in terms of agriculture we would be the primary the ones that we go there and apply for ethics approval”…IDI 01*.

#### Dilemmas from medical framing during the review of ANH research

The attitude of researchers towards the review systems differed. Some researchers referred to the heterogeneity in principles and practices that govern medical research with those that govern the ANH discipline. To expound on the issue, they explained that some practices, for example, issues of compensation and the clinical trial construction of confidentiality, brought in many dilemmas. The researchers explained that the compensation approach in the ANH field differs from that in medical research [24]. ANH researchers consider agriculture studies socially beneficial. Compensation may include the opportunity for communities to gain new knowledge or receive quality seed.

*“…In other communities where we do agriculture nutrition research, compensation is in a form, for example, seed. For example, we would go to an area and provide seeds because our interest is in the practices that we would do; improving dietary practices, infant nutrition, feeding, and then, if we get them, for example, soybeans and they grow, they would not get anything but maybe if they did not have good quality seed and after harvest, everything is there, and that is the compensation and they are giving us data, so we would compensate in that way. In other areas, compensation is that now they are more knowledgeable because we embedded nutrition and education, and you are leaving a far more knowledgeable community, more skilled than they were. I look at all that as a form of compensation in nutrition research.” IDI 01*.

Money compensation has been a source of concern for researchers, especially in ANH research. Incentives and compensation were heavily emphasized as factors that would impact research sustainability. Due to the fact that most nutrition studies are operational studies conducted in a community setting, researchers were concerned that monetary compensation would hinder the implementation of primary ANH interventions. Additionally, researchers were concerned that a medical model of compensation in ANH research would create an undesirable precedent for future research. “*….In operational research, you are mostly doing things in the garden and where the community live, so they already own whatever is happening, all you are bringing in is a new way of thinking and perhaps through the technology of doing things and also, l am glad you have mentioned the word sustainability because many partners would say it is not sustainable because you want to give them money or any other forms, not just money but any other forms of compensation for time.” IDI 01.*

Additionally, researchers noted that monetary compensation may be a significant barrier to implementing ANH interventions. The central concern here was the acceptability and ownership of any research project designed to address a specific public health concern.

“……*In terms of medical ethics and agriculture ethics, I would say there are differences because l remember during the trial there were issues to do with compensation, which is not promoted in agriculture because they want participants to own the agriculture program. …., in terms of sustainability, so if l would go to the field and compensate them, the next programme when it comes you don’t, the participants will not, in another programme they will not enter a programme because there is no element of compensation…,” IDI 02*.

“……*The question is if this technology works are you going to give money to people to do it.? So that becomes a concern, when we go to communities and work with them with nutrition projects they are part of it, and we are forming this partnership. As long as they understand that you are there to do research and you are not hiding anything and whether it works or not they are part of it and if it becomes something they can adopt, they do and if it is not going to work, you understand each other that this was not going to be the way that it is.* IDI 01.

#### Transparency and accountability within the system

The researcher’s collaboration with community members permits the conduct of ANH projects. Central to their cooperation is transparency and accountability in terms of the project’s objectives, relevance and participants’ roles and responsibilities. In addition to understanding project requirements and willingness to work with researchers, community members must also be actively involved in assessing the effectiveness of the intervention. The relationships are built on accountability and transparency, which support reliable and trusted processes. For ANH research to be owned and sustained, these processes and systems are essential.ANH approaches are developed more quickly and effectively through operational research. Community members’ inability to distinguish research projects from nutrition and health interventions and farm-based extension services may negatively affect the uptake of research activities beneficial to community wellbeing. Researchers were concerned that adopting a medical model of operationalising ethics, such as monetary compensation, may impede operational research. “….*And then we ask them for clarification. And they say your colleagues when we were involved in research they gave us some money so shall l be paid for measuring my child? So that is how bad things can get sometimes that you are going to a community, that is my opinion, that is a bad expectation, as much as you look at it in this way but someone is coming in to help you that you should monitor your child. You should monitor your situation and you should intervene quickly and then you are expecting to be paid. I think that has a bad compensation, people cannot draw a line, that just say ooh is it research and research we receive money and give us the money now. People do not look at it as being something different and that is there for them for their benefit, to be able to identify malnutrition at the earliest time and so that is how l look at it as bad things can go when we give compensation and we emphasise a lot on compensation and l do not see why we should compensate money.” IDI 01*.

Some researchers mentioned that ANH is a new nexus that is cutting across three dimensions; therefore, research ethics committee members must understand the variability of ANH research and stratify the review according to expert knowledge in health, nutrition and agriculture.

**‘’’***Sometimes l wonder the extent to which the national health science research ethics committee within the Ministry of Health reviews our applications when we send them there because all are valid and it’s everything within nutrition, everything with agriculture and a mixture of that. And l get worried and this is what l have been talking to our leaders in the directory of research and outreach and say let’s have an internal research ethics committee because it would have varied membership, people that can understand the issues that are there in pure agriculture, pure nutrition and then in between where these are mixed because there are quite varied, so that is one of my worries. IDI 01.*

### Overarching aspects and recommendations on ethics review practices

Researchers highlighted the need to build research ethics capacity in ANH research and establish research ethics review systems that complement existing systems in addition to provideing specific capacity for reviewing agricultural interventions.

*“…, From agriculture nutrition, we haven’t taken ethics seriously otherwise we had, let’s say for example; we haven’t yet had a body that deals with specifically research on agriculture research…. and nutrition we haven’t yet reached the point, for example, we have no officer who was trained to deal with all the research with other partners, we don’t have that and that alone we have to say we have enough of these issues, which I think we have to start building the capacity of our officers in terms of ethics. Even getting somebody at PhD or master’s level on ethics* in agriculture*” IDI 08*.

The researchers had diverging views on the appropriate form that the current ethical review process should take, and no consensus was reached.

*“….So, ah, if you just have only those that understand agriculture, they may not see the need. So, we were doing the micronutrients; micronutrients have an interaction. I would hope someone not in agriculture might not necessarily focus on that; you would want someone on the ethical board who did have some medical health knowledge to know what could have happened with micronutrients. It could not be good to have someone who has been medically trained because they wouldn’t necessarily know what would happen to agriculture and the environment; they wouldn’t know why they have that information. So, I guess they might not have an exceptional idea of how it would impact the participants. I mean, the people we were collaborating with were essentially farmers, so we might have this sort of excellent health outcome without really having the daily impact on their living. We needed to have someone who understands why these such interventions, what impact these interventions have on the outside the health response” IDI 09*.

Some researchers commented that it would be ideal for integrating ANH researchers in already existing REC. Others reported that financial resources might be a significant barrier in establishing fully-fledged ANH Research Ethics Committees (ANHREC).

***“….****the idea is to engage the clinical component and the Agricultural component, trying to get one understanding of both and how they’re interrelated and mapping the way forward from that. IDI 04*.

Researchers emphasized the importance of an independent ANHREC. They identified the NHSRC and institutional review committees (e.g., COMREC) as resources to promote ANH research ethics. However, there is no institutionally independent ANHREC. There were also restrictions regarding the ethical review of ANH research using the medical model. The absence of specialized ANHRECs made maintaining the values that govern ANH researchers difficultly. The medical model for ethics review raises various dilemmas due to conflicting values and prioritizations in the medical field and the ANH field, for example, issues of compensation. Table 2 provides an overview of researchers’ awareness, attitude, and practices regarding ANH research in Malawi.

**Table 2:** A description of ANH researchers’ awareness, attitude, and practices

	Awareness	Attitude	Practice
Leadership	• State the need for building ethical competence and moral reasoning. • Seen to be a lack of training initiatives supporting the needs of ANH researchers	• Promoting the importance of ethics education in ANH research and the ethical competence of ANH researchers	• Participate in an ethics discussion • Enrolling in ethics education. • Developing specialised ANH ethics education
Ethical Consideration	• Researchers acknowledged diverse ethical considerations for ANH research. • Awareness of ethical principles and values that govern the conduct of research	• Using general research frameworks from medical research can be challenging. • Highlighting that the current practice of operationalising clinical medical ethics in ANH research can raise wider ethics issues.	• Operationalisation of general ethical principles in ANH research. • Identifying conflicts when medical research values are operationalised for ANH research.
Governance	• Lack of specialised guidelines and research frameworks	• Currently utilising generic research guidance documents	• Recommending research frameworks relevant to the ANH field
Systems	• Identification of the system gap • Raised concerns about the dominance of medical-oriented systems	• No RECs in Agriculture, Nutrition and Health • Need for discipline relevant/specific RECs	• Developing ANH research review processes • Establishing new AHN RECs or expanding the capacity of medical research ethics review committees

## Discussion

To our knowledge, this is the first study to examine the perceptions and attitudes of leading scientists in the ANH field regarding the conduct and governance of agriculture, nutrition, and health research in Malawi. From different backgrounds, roles and responsibilities, the experiences and reflections of these ANH researchers raise several key topics and pose some important questions for research ethics and the ANH community. Several of the insights reported here are supported by published studies with researchers in other sectors, but others raise nuanced questions that have never been addressed before. The LEGS framework was originally proposed to support the strengthening of health systems by setting goals and pathways and as a tool to examine the status quo[19]. In this context, it has been a useful tool for the analysis of experiences and needs, in terms of ethics processes and capacities in ANH research.

### Leadership

Almost all researchers highlighted the importance of ethical leadership and governance, as well as the need for ethics training to underpin competence in ethics and support leadership development. Based on Grady (2008)’s study, education and training in ethics have a significant effect on healthcare workers’ confidence, use of ethical resources, and moral behaviour [25]. According to Ndebele [26]learning opportunities can enhance a better understanding of research ethics in low- and middle-income countries (LMICs). This study showed that ethics education was a prerequisite to enhancing ethical decision-making and supporting ethical competence. In our study, moral competence was defined as the awareness, knowledge, abilities, and attitudes necessary to address ethical issues [27, 28]. Researchers in this study were also able to acknowledge ethical challenges in the ANH field and expressed difficulties dealing with various dilemmas and complexities they faced. As part of their work as a broader trial team, researchers reflected on and emphasised the importance of dialogical interaction sessions, leading to ethical reflections and shared decision-making. Based on Hemberg and Hembergs’ work, reflective meetings enhance researchers’ ethical competence since they permit deliberations [14].

### Ethics

The participants emphasised the importance of some of the ethical implications of conducting ANH research, and mapping issues related to community participants. ANH research raised similar issues to all human participant research, which are addressed by existing national and international standards. Among these are preventing harm, respecting research participants, promoting autonomy, engaging with communities, and ensuring justice [29–31]. However, this study also revealed the need for a framework that guides ANH research implementation. The findings are in line with studies that have echoed the need to develop ethical frameworks that incorporate core ethical principles. Nevertheless, they support the development and implementation of agricultural, nutritional, and health interventions [32–34]. Due to the lack of holistic frameworks in some specific and emerging research fields, the biomedical profession model has been directly transferred and generally enjoyed popularity in the way ethical principles have been adopted over the decades [29].

Biomedical profession models have been utilised in emerging research fields, such as ANH, due to the lack of holistic frameworks [29]. However, the unreflective application of medical disciplines can create dilemmas if not re-examined. It can be helpful to discuss the underlying principles and values with participants, stakeholders and researchers before they are operationalised in other disciplines. The operationalization of ethical principles requires careful attention and consideration, as different approached to weighing ethical dilemmas may lead to different decisions and outcomes [5]. The study found that agriculture is a practice and profession that upholds a range of values. Agricultural reimbursements, for example, are operationalised differently than medical reimbursements. To promote ownership, acceptability, and sustainable benefit sharing, agriculture refrains from providing monetary compensation, whereas in medicine monetary compensation is used to cover individual participants’ travel expenses, lost time, and medical expenses [24]. These payment issues are argued not to be feasible for agricultural research, but what matters is not whether they are feasible but whether they undermine ethically relevant aspects of the relationship, benefit-sharing values, societal values, and other aspects of agricultural research and related ANH research. ANH research should therefore use ethical values as a moral compass to assist in problem-solving and decision-making.

### Governance

Researchers in this study demonstrated their knowledge of policies and guidelines that govern the conduct of research in Malawi. Progress has been made in developing adapted guidance in the area of health research but there were concerns that the procedures are not well adapted and even operationalised to meet the needs of specialised and linked research areas, such as animal agriculture research and ANH research cutting across the three dimensions. There was a perceived lack of support mechanisms and specialised guidance.

The study findings that the researchers indicate that current research legislative and guidelines have not kept pace with trends in large scale multi-partner and multi-disciplinary research, such as those projects that site within the ANH nexus. The gaps in tailored research guidelines may hamper the promotion of ethical research conduct in well-established fields like agriculture and the new nexus of ANH research. Drawing lessons from work in outer areas could be valuable in this context as demonstrated by a study by Whitworthwho reported thatthat restrictive and non-progressive legislative architecture poses a huge barrier to advancing health research in Africa [10]. This study did not explore the applicability of research guidelines to the ANH field, but it did identify numerous values that should be considered when operationalizing the principles that govern human research.While research activities have grown in the past two decades [35] researchers observed that Malawi’s research guidelines require significant improvement.

### Systems

It is acknowledged that many ANH projects are reviewed by the National Research Ethics Review Board and Institutional Review Committees like the COMREC. Researchers in Malawi reported that institutions involved in agriculture and nutrition research do not have RECs. Divergent views appear on the need for an institutional review committee, with some arguing for better integration of RECs, strengthening capacities, and providing supporting protocols, while others argue for specialized ANHRECs.Despite the medical model of ethics review currently governing ANH research, researchers recommended reviewing, adapting, and developing this model to advance research values in ANH research. Although the availability of RECs promotes scientific and ethical conduct of research, it would take researchers in the ANH professions to step forward and develop greater skills in the ethics of ANH research to achieve adequate scientific and ethical review excellence. According to Dyer and Demeritt’s study [36], based on Dyer and Demeritt’s study, they questioned whether a one-size-fits-all approach would be appropriate for all disciplines. Furthermore, they emphasized that each field is legitimately guided by a variety of values, principles, and practices. Introducing heterogeneity into research practices and values raises ethical concerns due to conflicting values between different professions. Interviewees’ arguments for developing a specialised ANHREC may be explained by this gap. Nabyonga-Orem in their review of research governance in Africa, emphasized the importance of ensuring RECs represent relevant expertise and functionality [6].

### Limitations

A limitation of this study is the recruitment and interview process. Since the bioethics team conducting and analysing the interviews was part of the ANH project, bias might be introduced due to familiarity. However, familiarity was a strength as the BT selected respondents who were appropriate and able to provide valuable and rich data for the study. They also permitted respondents to freely share their insights and perspectives on the project as a whole. The work presented here did not focus on evaluating RECs that oversee research implementation in Malawi.Furthermore, since only one trial is included in this study, it may not offer a comprehensive picture of the ethical issues that surround ANH research. To develop a framework for ANH research, future studies should examine a diverse range of studies and their specific ethical issues.

### Conclusions and recommendations

Building ethical competence, leadership, and systems is essential to enhancing the ethical conduct of ANH research. Researchers in community-based ANH research can promote ethical mindfulness if they have the necessary knowledge, skills, and attitudes. In the study, it was found that there is an emergence of a multi-partner programme within ANH that may impact various aspects of research ethics. Conducting and reviewing ANH research might require redefining values. Additionally, the analysis of the moral complexity of applying a medical ethics model to agriculture-related research needs to be further investigated.

To provide practical guidance to agriculture professionals and define values for ANH research, a framework of ethics analysis specifically tailored to the field of ANH is needed. Therefore, future research should explore values that inform ANH research, as well as assess the ethics education needs of ANH researchers. Research could also be conducted to develop ANH researchers’ readiness and capabilities in recognizing and responding to ethical issues that arise in research. As a result, ANH researchers would be able to handle ethical issues in their everyday research practice.

### Electronic supplementary material

Below is the link to the electronic supplementary material.

**Additional File 1:** KI Agriculture Version 1.0 dated 08 October, 2019

## Data Availability

The data that support the findings of this study are available from CEBESA but restrictions apply to the availability of these data, which were used under license for the current study, and so are not publicly available. Data are however available from the authors upon reasonable request and with permission of CEBESA. Contact the primary author Ms Limbanazo Matandika.

## List of abbreviations

AHHA: Addressing Hidden Hunger with Agronomy, ANH RESEARCH:Agriculture, Nutrition, and Health Research
ANH: Agriculture, Nutrition and Health
COMREC: College of Medicine Research Ethics Committee
FGD: Focus Group Discussion
IDI: In-depth interview
LEGS: Leadership, Ethics, Governance and Systems
LUANAR: Lilongwe University of Agriculture and Natural Resources
NHSRC: National Health Sciences Research Ethics Committee
REC: Research Ethics Committee
BT: Bioethics Team
TP: Trial Participant
RTREA: Real-Time Research Ethics Approach
AHHA: Addressing Hidden Hunger with Agronomy
ANH: agriculture-nutrition-health
TImT: Trial Implementing Team

## References

[1] Chilimba ADC (2011). Maize grain and soil surveys reveal suboptimal dietary selenium intake is widespread in Malawi. Sci Rep.

[2] Hurst R (2013). Soil-type influences human selenium status and underlies widespread selenium deficiency risks in Malawi. Sci Rep.

[3] Phiri FP (2019). The risk of selenium deficiency in Malawi is large and varies over multiple spatial scales. Sci Rep.

[4] Tom JFC, Beauchamp L (2012). Principles of Biomedical Ethics.

[5] Kruger M, Ndebele P, Horn L. *Research Ethics: A Resource for Research Ethics committees*. 2014. doi: 10.1017/CBO9781107415324.004.

[6] Nabyonga-Orem J, Asamani JA, Makanga M (2021). The state of health research governance in Africa: what do we know and how can we improve?. Heal Res Policy Syst.

[7] Msoroka MS, Amundsen D (2018). One size fits not quite all: Universal research ethics with diversity. Res Ethics.

[8] Kass NE (2007). The structure and function of research ethics committees in Africa: a case study. PLoS Med.

[9] Kasule M, Wassenaar DR, Ijsselmuiden C, Mokgatla B (2016). Silent voices: current and future roles of african research ethics committee administrators. IRB Ethics Hum Res.

[10] Whitworth JA (2008). Strengthening capacity for health research in Africa. Lancet.

[11] Vanclay F, Baines JT, Taylor CN. “Principles for ethical research involving humans: Ethical professional practice in impact assessment Part I,” *Impact Assessment and Project Appraisal*, vol. 31, no. 4. Taylor & Francis, pp. 243–253, 2013. doi: 10.1080/14615517.2013.850307.

[12] Falkenström AT, Ohlsson E, Höglund J. “Developing Ethical Competence in Healthcare Management,” 2016.

[13] Höglund AT, Helgesson G, Eriksson S (2010). Ethical dilemmas and ethical competence in the daily work of research nurses. Heal Care Anal.

[14] Hemberg J, Hemberg H (2020). Ethical competence in a profession: Healthcare professionals’ views. Nurs Open.

[15] Baumgarten E (2015). The concept of ’ competence " in medical ethics. J Med Ethics.

[16] Pettersson M, Hedström M, Höglund AT (2018). Ethical competence in DNR decisions -a qualitative study of swedish physicians and nurses working in hematology and oncology care. BMC Med Ethics.

[17] Joy EJM, et al. Can selenium deficiency in Malawi be alleviated through consumption of agro-biofortified maize flour? Study protocol for a randomised, double-blind, controlled trial. Trials. Dec. 2019;20(1):1–9. 10.1186/S13063-019-3894-2/FIGURES/2. 10.1186/s13063-019-3894-2 PMC6937860 31888766

[18] Hurlimann T, Peña-Rosas JP, Saxena A, Zamora G, Godard B (2017). Ethical issues in the development and implementation of nutrition-related public health policies and interventions: a scoping review. PLoS ONE.

[19] Mfutso-Bengo J, Kalanga N, Journal EM-B-MM. and U. 2017, “Proposing the LEGS framework to complement the WHO building blocks for strengthening health systems: One needs a LEG to run an ethical, resilient system for,” *Ajol.Info*, vol. 29, no. December, pp. 317–321, 2017, doi: 10.4314/mmj.v29i4.7. 10.4314/mmj.v29i4.7 PMC6019549 29963287

[20] Joy EJMM (2019). Can selenium deficiency in Malawi be alleviated through consumption of agro-biofortified maize flour? Study protocol for a randomised, double-blind, controlled trial. Trials.

[21] Joy EJM (2019). Can selenium deficiency in Malawi be alleviated through consumption of agro-biofortified maize flour? Study protocol for a randomised, double-blind, controlled trial. Trials.

[22] Creswell JW, “, Creswell JW. (2003). Chapter One, ‘A Framework for Design.,’” *Res. Des. Qual. Quant. Mix. methods approaches*, pp. 3–26, 2003, doi: 10.3109/08941939.2012.723954.

[23] Braun V, Clarke V. “Using thematic analysis in psychology,” 2006.

[24] Gordon SB, Chinula L, Chilima B, Mwapasa V, Dadabhai S, Mlombe Y (2018). A Malawi guideline for research study participant remuneration. Wellcome Open Res.

[25] Grady C, Danis M, Soeken KL, Donnell PO, Taylor C, Ulrich CM (2009). “NIH Public Access.

[26] Ndebele P et al. “Review of NIH Fogarty-Funded Programs 2000–2012,” vol. 9, no. 2, pp. 24–40, 2014, doi: 10.1525/jer.2014.9.2.24.Research. 10.1525/jer.2014.9.2.24 PMC4280231 24782070

[27] Koskenvuori J, Stolt M, Suhonen R, Leino-Kilpi H (2019). Healthcare professionals’ ethical competence: a scoping review. Nurs Open.

[28] U. of T. H. S. C. in S. A. Robichaux, Catherine, *Ethical competence in nursing practice. Competencies, skills, decision making*, First Edit. New York,: Springer Publishing Company LLC, 2016. [Online]. Available: https://www.springerpub.com/ethical-competence-in-nursing-practice-9780826126375.html#productdetails.

[29] Tom JFC, Beauchamp L, Tom JFC, Beauchamp L (2001). Principles of Biomedical Ethics.

[30] Emanuel EJ, Wendler D, Killen J, Grady C. “What Makes Clinical Research in Developing Countries Ethical ? The Benchmarks of Ethical Research,” vol. 189, pp. 32–34, 2004. 10.1086/381709 14976611

[31] Grady C (2004). Ethics of vaccine research. Nat Immunol.

[32] Kass N, Hecht K, Paul A, Birnbach K (2014). Ethics and obesity prevention: ethical considerations in 3 approaches to reducing consumption of sugar-sweetened beverages. Am J Public Health.

[33] Riiser K, Løndal K, Ommundsen Y, Misvær N, Helseth S (2015). Targeting and tailoring an intervention for adolescents who are overweight: some ethical concerns. Nurs Ethics.

[34] Have MT (2014). Ethical aspects of obesity prevention. Best Pract Res Clin Gastroenterol.

[35] Mungwira RG (2015). Is it ethical to prevent secondary use of stored biological samples and data derived from consenting research participants? The case of Malawi Ethics in Public Health, medical law, and health policy. BMC Med Ethics.

[36] Dyer S, Demeritt D (2009). Un-ethical review? Why it is wrong to apply the medical model of research governance to human geography. Prog Hum Geogr.

