# Supplementary material for: Perspectives of agriculture, nutrition and health researchers regarding research governance in Malawi. Using a leadership, ethics, governance and systems framework
Source: BMC Med Ethics. 2023 Aug 21;24:66. doi: 10.1186/s12910-023-00940-x (PMC10441702; doi:10.1186/s12910-023-00940-x)
Supplement: Supplementary file 1 — Additional File 1: KI Agriculture Version 1.0 dated 08 October, 2019 [file 12910_2023_940_MOESM1_ESM.docx]

**Leadership**

1. Would you please tell me about yourself, including your current role in the recent study?
   - **Prompt:** How long have you been working at the current role? What s/he enjoys about the role especially relationship with community members ?
   - **What projects/research activties/interventions are you currently implementing and where.**
   - Probe about their role in line with governement and community agenda.
2. What do you consider important about your job/role in this tudy?
3. What do you know about research ethics?
   - Probe about trainning, knwolegde on how to promote ethical conduct of research with human subjects
   - Relevance of ethics in agricutlure and nutrition research
4. dd

**Trial experiences and relflections**

1. How would you describe your overall trial experience from the time this project commenced?

- Probe about inovlement in protocol design, formative research, trial activties, workshops, involevement with trial participants in Kasungu

1. What has been your overall expeience during the conduct of this trial?
   - Probe what made theis research experience difference from any other research experience?
   - Probe about the involvement of the ethics team
2. How would you describe your experience with the ethics team in agricutlure, nutrition and health research project?
   - Probe about benefits and challenges
3. **Ethics**
4. What values inform the implementation of Agricultutue and nutrtion research/ interventions?
5. What values do you think uphold Agriculture research?
   - Probe: about its impact? How do they measure impact?
   - What are some of the outcomes of this approach?
6. What were some of your responsibilities as a reseachers in this trial?
7. How would you describe your understanding of ethics in agriculture
   - Probe: what are some of the ethical issues that youy encountered in the research?
   - Probe:How would you narrate your over expoure to ethics? Trainning, teachings and programme/project support

**Reseearch Governance and systems**

1. What are the structures, processess and pratices that support the conduct of Agrictulture and nutrition research in Malawi?
   - Probe: How are research proposals reviewed? By who?
   - What stakeholders are involved, their mandate and role in the governance system
   - Are there guiding principles that inform the research practice?
   - Probe about strategies that imporove the conduct of research
   - What kind of policy is used in Agriculture to govern the conduct of research?
   - How do they researchers in agriculture safeguard the interest of those particpatin in research
   - Their benefits to current Malawian setting?
   - Probe for any guidelines or reulations that govern agriculture, nutriton an health research
     - Probe: Their relevance, availbility, applicability
   - Challenges and advantages of this approach
   - Recommendations and suggestion for solving the challenges
2. How would you describe the importance of structures, procedures and processes that promote ethical conduct of research in agricutlre?
   - - Probe: How the pricniples were applied,
3. THIS IS THE END OF THE QUESTIONNAIRE:
4. REMEMBER TO THANK THE RESPONDENT FOR THEIR TIME.
